# Multiscale modelling of cerebrovascular injury reveals the role of vascular anatomy and parenchymal shear stresses

**DOI:** 10.1038/s41598-021-92371-0

**Published:** 2021-06-21

**Authors:** Siamak Farajzadeh Khosroshahi, Xianzhen Yin, Cornelius K. Donat, Aisling McGarry, Maria Yanez Lopez, Nicoleta Baxan, David J. Sharp, Magdalena Sastre, Mazdak Ghajari

**Affiliations:** 1 Dyson School of Design Engineering, Imperial College London, London, UK; 2 Shanghai Institute of Materia Medica, Shanghai, China; 3 Department of Brain Sciences, Imperial College London, London, UK; 4 Biological Imaging Centre, Imperial College London, London, UK; 5 Centre for Blast Injury Studies, Imperial College London, London, UK

**Keywords:** Biomedical engineering, Computational models

## Abstract

Neurovascular injury is often observed in traumatic brain injury (TBI). However, the relationship between mechanical forces and vascular injury is still unclear. A key question is whether the complex anatomy of vasculature plays a role in increasing forces in cerebral vessels and producing damage. We developed a high-fidelity multiscale finite element model of the rat brain featuring a detailed definition of the angioarchitecture. Controlled cortical impacts were performed experimentally and in-silico. The model was able to predict the pattern of blood–brain barrier damage. We found strong correlation between the area of fibrinogen extravasation and the brain area where axial strain in vessels exceeds 0.14. Our results showed that adjacent vessels can sustain profoundly different axial stresses depending on their alignment with the principal direction of stress in parenchyma, with a better alignment leading to larger stresses in vessels. We also found a strong correlation between axial stress in vessels and the shearing component of the stress wave in parenchyma. Our multiscale computational approach explains the unrecognised role of the vascular anatomy and shear stresses in producing distinct distribution of large forces in vasculature. This new understanding can contribute to improving TBI diagnosis and prevention.

## Introduction

Traumatic brain injury (TBI) is an important worldwide public health issue and the leading cause of death and disability in young adults^1–5^. More than 10 million TBI cases worldwide occur every year resulting in hospitalization or death^6^. TBI is initiated by mechanical forces leading to primary injuries, including vascular damage^7–10^. From this, secondary injuries follow, such as neuroinflammation. The spatio-temporal complexity of the pathophysiological response after TBI makes the prediction of the clinical outcome difficult^11^. More importantly, it is not well understood how mechanical forces damage vessels, while this understanding is key to the prevention of vascular injuries through better design of protective measures, such as helmets.

The immediate and early effects of TBI on the vasculature include haemorrhage and blood–brain barrier (BBB) disruption, later followed by hypoperfusion, altered delivery of metabolic substrates and hypoxic and ischemic tissue damage^7,12^. These changes lead to neurological dysfunction and accelerate disease development, which can result in behavioural deficits and neurodegeneration^12,13^. Previous neuroimaging and histopathology studies have shown heterogenous distribution of vascular injuries across length scales, from large vessels to capillaries^11,14^. Interestingly, post-mortem analyses of TBI victims have found intact vessels in the vicinity of abnormal vessels^15,16^. This raises a key question: how does the stress wave from the initial mechanical insult cause different force distribution and subsequent pathology in neighbouring vessels? We hypothesise that the anatomy of vasculature plays a key role in determining the distribution of mechanical forces in vessels.

Finite element (FE) modelling can be used to test this hypothesis, as it allows us to predict mechanical forces in fine anatomical features of the brain with sufficiently high temporal resolution required for mechanical loading^17^. Several finite element models of TBI have been developed to predict mechanical forces in the brain tissue, using different measures, e.g. stress (a measure of local force), strain (a measure of local deformation) and strain rate. A few models were tested against experimental TBI in vivo, comparing the distribution of stress with patterns of pathology in animal models^18–21^. For example, a recent study has shown that the distribution of the FE predicted axonal deformations correlate with the locations of acute traumatic axonal injury in a pig model^22^. Another study showed that FE modelling can predict rupture of bridging veins in the pig model^23^. In our recent paper, we showed that computational modelling of TBI in rats predicts patterns of post-traumatic cortical lesions and glial and axonal injuries^24^. However, these models lack a description of cerebral vasculature, which limits their ability to predict patterns of vascular injury seen after TBI. In particular, a multiscale explicit model of the vasculature is needed to test whether there is an interaction between vascular anatomy and forces in the parenchyma across length scales in the short time of head loading.

To address this question, we incorporated a detailed definition of the cerebral vasculature, obtained from high resolution synchrotron imaging, into a rat finite element model of TBI. We modelled our injury based on the Controlled Cortical Impact (CCI) model, an established TBI model that reproduces key pathological features of human TBI including BBB disruption^25,26^, and predicted stress in vessels and the surrounding tissue. The axial stress in vessels was used as a primary measure, as it is likely to be the main cause of vascular damage^27^. In order to accurately predict axial stress in the capillaries, we developed microscale models of the regions exposed to large deformations. We examined our hypothesis that the stress wave propagated within the brain tissue interacts with the anatomical structure of the vasculature to produce a complex distribution of axial stress in the vascular network. We also tested whether the stress and strain in the parenchyma are correlated with the axial stress in vessels. To validate the model predictions of tissue and vascular damage, we performed CCI in rats, mapped patterns of BBB damage post-injury and compared these with the distribution of large stresses in vessels.

## Results

### Large axial stress in vessels at the locations of fibrinogen extravasation

**Figure 1:**
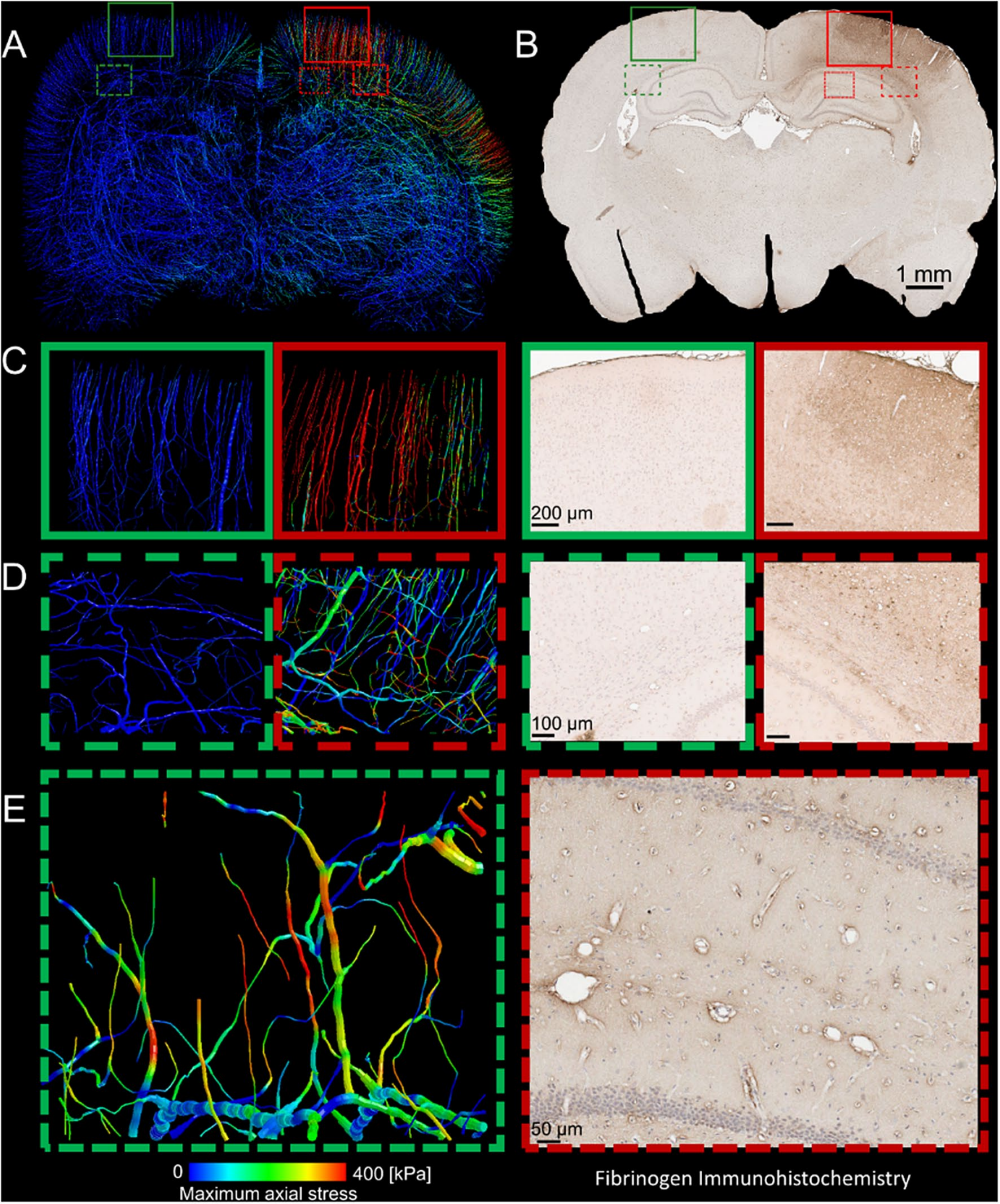
Computational modelling predicts Fibrinogen extravasation. (**A**) The axial stress distribution within the vasculature, (**B**) representative whole brain photomicrograph of sections from animals subjected to 2 mm CCI (3 days post injury) at − 3.12 mm from bregma stained for Fibrinogen. Computational prediction of maximum axial stress and corresponding Fibrinogen staining in (**C**), the contra- (green) and ipsilateral (red) cortex, the contra- (green) and ipsilateral (red) Corpus Callosum, (**D,E**) at the microstructural level of the hippocampus.

We compared the distribution of the axial stress in vessels with the distribution of BBB damage 72 h post-injury. Fibrinogen is a blood-borne molecule involved in coagulation cascade and its extravasation into the parenchyma is a sign of vascular injury as a result of greater BBB permeability. Maximal fibrinogen extravasation was noted across the ipsilateral hemisphere, with the greatest fibrinogen extravasation below the impact site and near the edges of the impactor, where we predicted large stress in vessels (Fig. 1A,B). Fibrinogen extravasation generally extended towards deeper cortical layers, corpus callosum (Fig. 1D) and hippocampus (Fig. 1E), with notable leakage from penetrating vasculature (Fig. 1A–C). We also predicted large stress in the vessels located in these anatomical regions. Macroscopically, the contralateral hemisphere showed little evidence of fibrinogen extravasation, as was predicted by the computational model (Fig. 1C,D). Microscopic examination showed no evident extravasation throughout the neocortex, corpus callosum or hippocampus, with positive fibrinogen staining outlining intact vasculature. We predicted small stresses in these regions.

### Strong correlation between fibrinogen extravasation and large stress in vessels

**Figure 2:**
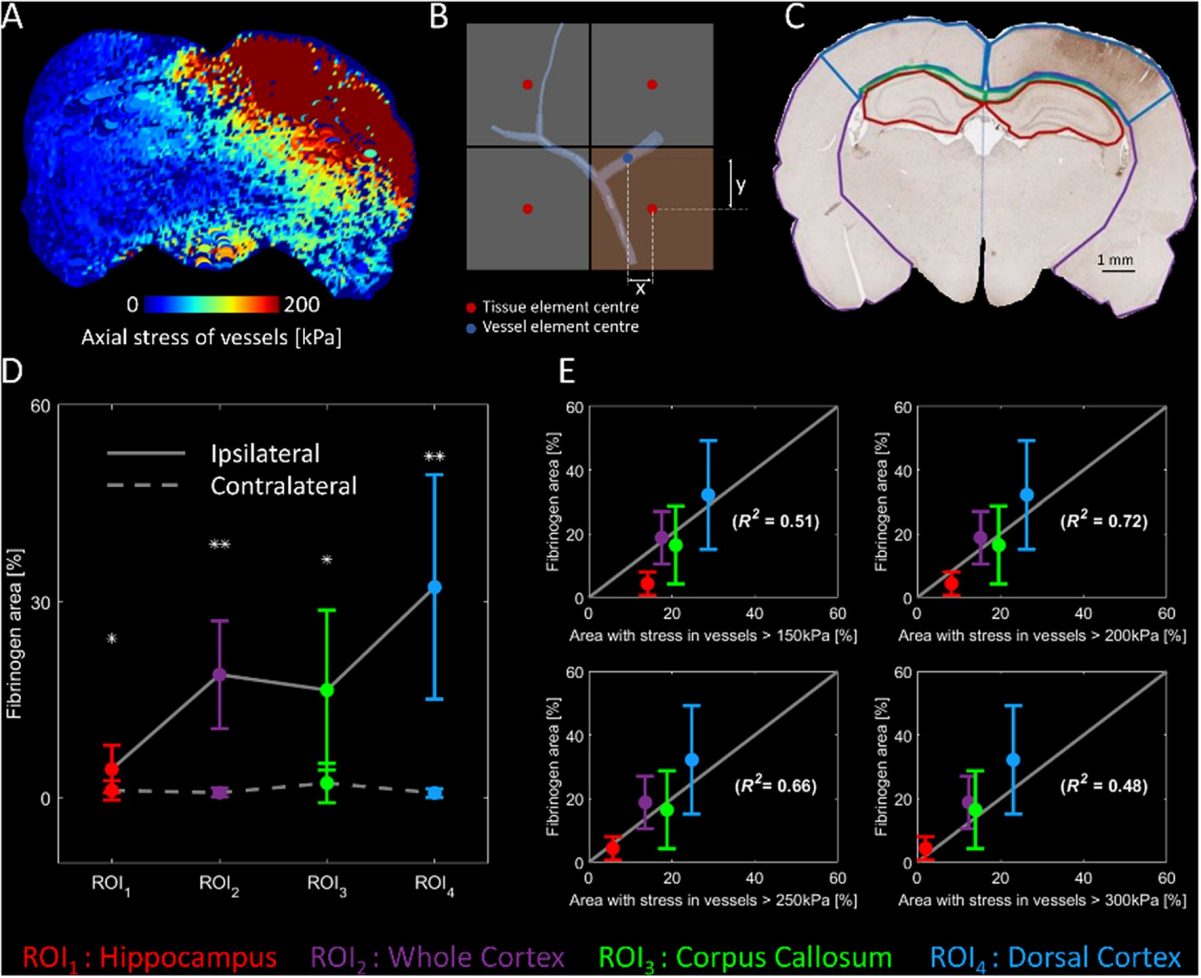
Quantitative comparison of numerical simulation and experiments. (**A**) Vessels’ axial stress mapped on the brain tissue. (**B**) For quantitative comparison between the fibrinogen extravasation and the stress in the angioarchitecture, stress of beam elements of the vasculature in the domain of tissue elements are mapped to the corresponding solid element. (**C**) Four segments of the brain tissue used for quantifying fibrinogen extravasation. (**D**) The fibrinogen extravasation is significantly higher in the ipsilateral hemisphere (* and ** indicate p < 0.05 and p < 0.005 respectively). (**E**) Correlation between the vessels’ axial stress and fibrinogen extravasation [error bars in (**D,E**) showing the standard deviation of the results among all tested animals].

In order to make a quantitative comparison between the extent of fibrinogen extravasation and the area of vessels undergoing large axial stresses, we mapped axial stress of vessels onto the brain tissue. The maximum axial stress of the vessels located in the neighbourhood of each element of the tissue was determined and assigned to that element (Fig. 2A,B). We used this map to determine the area of the brain tissue where axial stress in the vessels exceeded 150, 200, 250 and 300 kPa values across four regions of interest (ROI): corpus callosum, hippocampus, dorsal cortex and whole cortex (Fig. 2C). In order to quantify Blood–Brain barrier damage, the area of fibrinogen extravasation was measured in these ROIs.

For fibrinogen extravasation, repeated measures ANOVA with ROI and hemisphere as factors revealed significant main effects of ROI [F(2,15) = 9.2, p = 0.004] and hemisphere [F(1,9) = 12.0, p = 0.007] and an interaction between them [F(1,13) = 9.7, p = 0.005]. Post-hoc tests indicated that fibrinogen extravasation was significantly higher in ipsilateral ROIs compared to contralateral (Fig. 2D). The maximum extravasation was seen in the ipsilateral dorsal cortex.

A linear regression analysis between the areas with large vascular stress and fibrinogen extravasation in the ipsilateral ROIs showed that the correlation depends on the stress threshold (Fig. 2E). We found a strong correlation (R^2^ = 0.72, p < 0.003) between the area of fibrinogen extravasation and the area of the brain where vessels’ axial stress exceeded 200 kPa (equal to 14% axial strain considering the linear elastic material model used for vessels).

### Microscale modelling reveals profound differences in stress distribution in adjacent vessels

**Figure 3:**
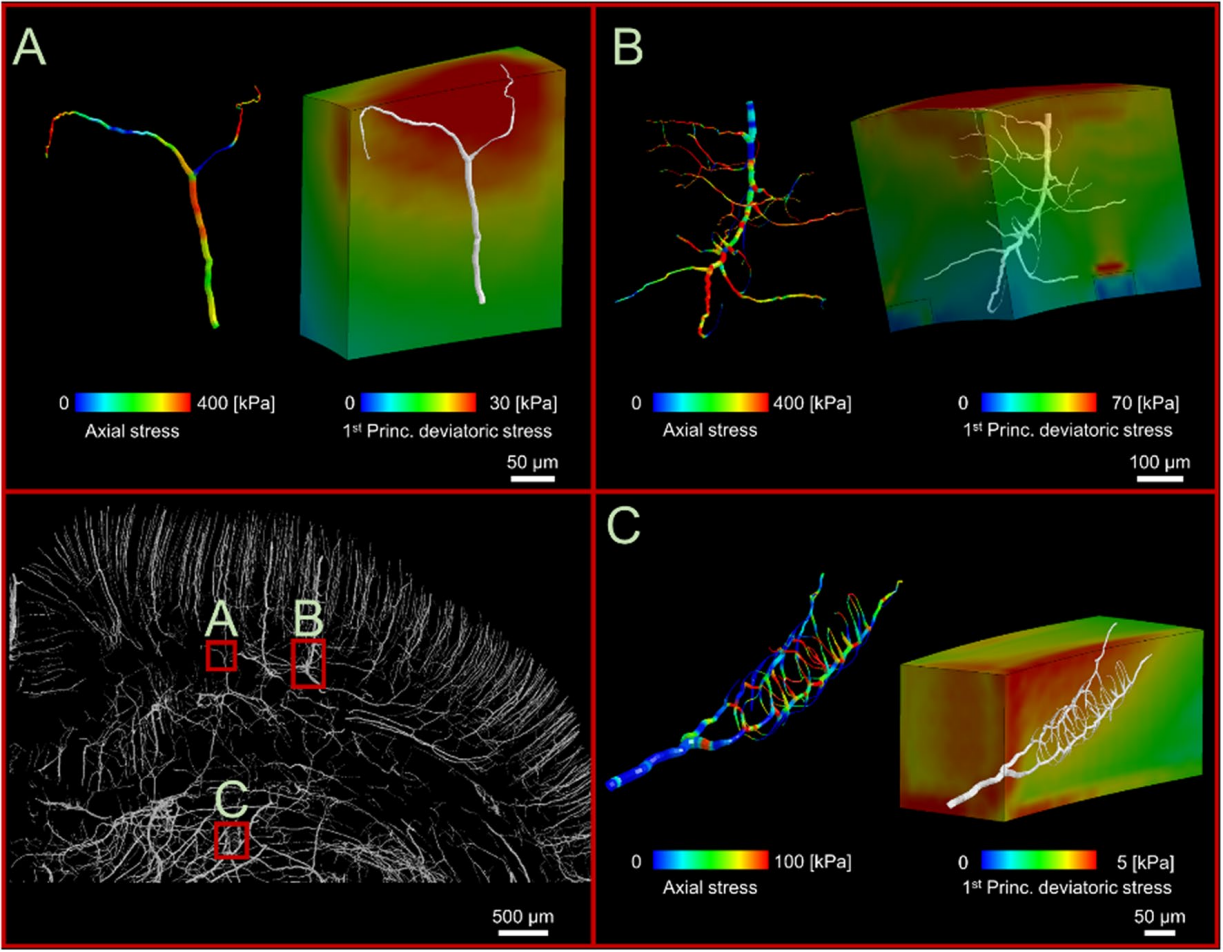
Examples of stress alteration at vessels which are only few microns apart. (**A**) Three different stresses in three branches of a bifurcation point show how the direction of vessels may result in three different levels of vascular stretching. (**B**) Capillaries branching out from a vessel with non-uniform stress distribution due to propagation of capillaries in different directions, (**C**) Capillaries with different stresses even though they are branching out from the same vessels and bifurcation points.

Our macroscale model predicts large variation in stress in vessels near the impactor (Fig. 1A). Our microscale models also reveal a patchy distribution of large stresses in vessels, even in areas where the stress is uniformly distributed in the parenchyma (Fig. 3). For instance, Fig. 3A shows that two vessels branching from the same bifurcation point are predicted to sustain different stresses, with one under large tension and the other not bearing any tensile force. Figure 3B shows nonuniform stress distribution across the length of a vessel, with concentrations near the bifurcation points, and Fig. 3C shows that neighbouring capillaries are predicted to sustain substantially different stresses. The patchy distribution of stress in vessels is the likely result of the interaction between the direction of vessels and the direction of maximal stress in the surrounding tissue.

### Vessels aligned with the direction of stress in tissue sustain larger stresses

**Figure 4:**
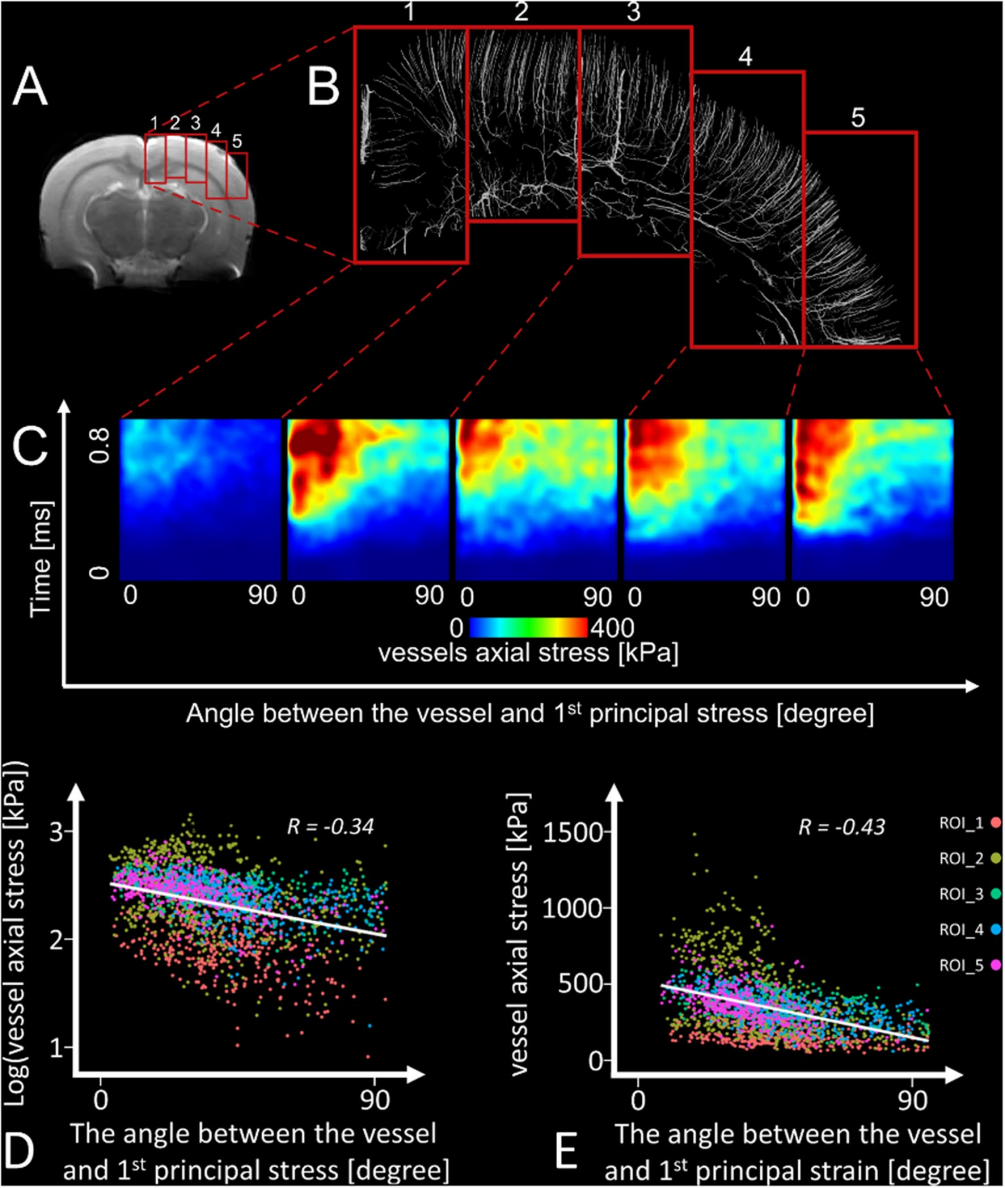
Microscale simulation for 5 ROIs. (**A**) The location of regions of interest. These regions of interest are in the ipsilateral hemisphere of the brain (cortex and corpus callosum) below the impact where the model predicts the highest strain and shear stress (Fig. 1), (**B**) the magnified view of the vessels within the regions of interest, (**C**) vessels’ axial stress as a function of time and the angle between the vessels and the direction of the 1﻿st principal stress of the neighbouring tissue. Vessels axial stress raises as the time passes and as the angle between the vessel and direction of 1st principal stress of the vessels’ neighbouring tissue goes towards zero. (**D**) Linear regression between the logarithm of the vessels’ axial stress and the angle between the vessels and the direction of the 1st principal stress. (**E**) Linear regression between the vessels’ axial stress and the angle between the vessels and the direction of the 1st principal strain.

We explored the interaction between the direction of vessels and the direction of maximal stress in their surrounding tissue in the ipsilateral cortex and corpus callosum, where large stresses and substantial BBB damage were observed (Fig. 1). The region was split into five segments and a micro-scale model was generated for each segment, allowing us to model very small vessels (Fig. 4A,B). The angle between the direction of the vessel and first principal stress in its neighbouring tissue was calculated. Figure 4C shows that when this angle tends to zero, i.e. when the vessel and stress direction align, the tensile stress in vessels increases substantially. This was confirmed by the Pearson’s correlation analysis, which showed a significant negative correlation between this angle and the tensile stress in vessels (Fig. 4D, R =  − 0.34, p < 0.001). This effect was slightly stronger when we controlled for the stress in the parenchyma (R_partial_ =  − 0.42, p < 0.001).

We extended our analysis to the strain in the tissue. We found a significant negative correlation between the tensile stress in vessels and the angle between the vessel and the direction of the parenchymal strain (Fig. 4E, R =  − 0.43, p < 0.001), with stronger correlation when controlling for the parenchymal strain (R_partial_ =  − 0.61, p < 0.001).

Vessels can undergo tension or compression, which is likely to be dependent on the angle between their axis and the direction of parenchymal stress. The receiver operating characteristic (ROC) curve confirmed that this angle can accurately predict whether vessels are under tension or compression (area under the curve (AUC) of 0.94 [95% CI 0.93–0.95]). Similar results were obtained for the angle between the vessel and the direction of the parenchymal strain (AUC of 0.95 [95% CI 0.94–0.95]).

### Correlation between stress in vessels and stress and strain in the parenchyma

**Figure 5:**
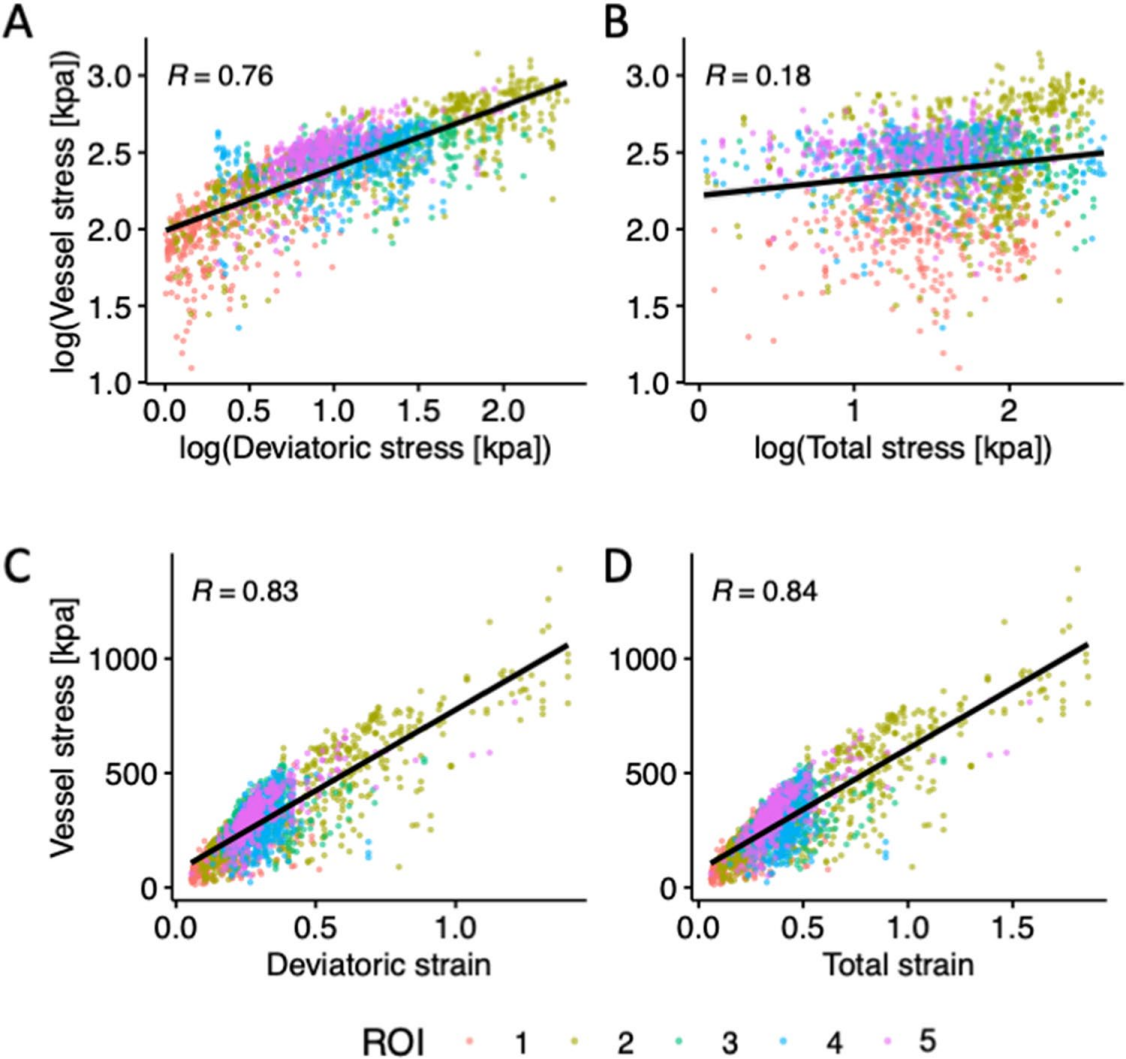
Correlation analysis for stress in vessels and (**A**) deviatoric stress, (**B**) total stress, (**C**) deviatoric strain and (**D**) total strain in the surrounding tissue. The data are predicted by the microscale models of vascular injury, developed for the five ipsilateral ROIs. Vessel stress refers to the tensile stress in vessels. Total stress and strain refer to the first principal stress and strain in the brain parenchyma and the deviatoric stress and strain refer to their deviatoric (shear) component.

In addition to the direction, the magnitude of the parenchymal stress is likely to increase the stress in vessels. It is also likely that the total and deviatoric (shear) stresses in the parenchyma have different effects on the tension in vessels. Hence, we tested whether the first principal stress in the tissue or its deviatoric component better correlates with the tensile stress in vessels. Pearson’s correlation analysis showed that the tensile stress in vessels has a strong positive correlation with the deviatoric stress in the neighbouring tissue (Fig. 5A, R = 0.76, p < 0.001). Similar results were obtained when controlling for the angle between the vessel and the direction of stress in the tissue (R_partial_ = 0.78, p < 0.001). There was however a weak correlation between the stress in vessels and the total stress in the surrounding tissue (Fig. 5B, R = 0.18, p < 0.001), with similar results when controlling for the angle (R_partial_ = 0.20, p < 0.001).

We extended our analysis to investigate the correlation between total and deviatoric strains in the tissue and tensile stress in vessels. The analysis showed strong correlations for both deviatoric (Fig. 5C, R = 0.83, p < 0.001) and total strains (Fig. 5D, R = 0.84, p < 0.001), showing that increasing both total and shear strains in the parenchyma increases stress in vessels. Similar results were obtained when controlling for the angle between the vessel and the direction of tissue strain (R_partial_ = 0.88, p < 0.001).

## Discussion

This study shows that during head impacts vessels can sustain large forces due to 1. large shear stresses in the parenchyma and 2. the alignment between the vessel and parenchymal stress. Our analysis was made possible by using a multiscale finite element model of vascular injury, which for the first time, incorporated a detailed definition of angioarchitecture in microscale. The refined rat brain model contains vessels down to a radius of 5 µm, which allowed us to predict stress distribution in small vessels that form the majority of the vascular structure^28,29^ and where key vascular pathologies, e.g. BBB breakdown and tauopathy, have been observed^30–32^. This approach reveals the key effect of the interaction between the vascular anatomy and mechanical forces in the parenchyma, providing a biomechanical explanation for the heterogenous distribution of pathology in vessels that are adjacent or branch from the same bifurcation point.

We developed a novel multiscale model of vascular injury biomechanics, which links tissue deformation across macro and micro scales. This approach allowed us to determine brain regions that undergo large deformation during the injury, develop microscale models for these regions, which incorporate details of microscopic vessels, and predict force distribution in the vessels. Previous studies incorporated vasculature in finite elements models of TBI in order to study the effect of the presence of the vasculature on brain deformation. These models incorporated large arteries and veins due to the low-resolution of the MR and CT images^20,21,33^. Here we used synchrotron imaging of brain slices to map fine details of the vascular anatomy^29^. A limitation of this approach however is that veins and arteries cannot be separated. Other methods based on using tissue clearing and machine learning have been recently proposed, which may help address this limitation in future work^28^. We used an image of the rat brain vasculature, which to the best of our knowledge, has the highest resolution among available images^29^. However, it may still have missed details of some of the smallest capillaries, leading to potential uncertainties in the model predictions. The effects of imaging resolution on the predicted stress and strains in very small capillaries should be addressed in future by using higher resolution images of the vasculature.

The predictions of the finite element model agreed well with the distribution of pathologies from our CCI empirical model. We found good agreement between the patterns of tensile stress in vessels and fibrinogen extravasation 3 days post-injury, when the BBB abnormality reaches its peak^25,34^. This provides initial validation for the model prediction of vascular injury and that tensile stress in vessels can predict BBB damage. We found strong correlation between the area of Fibrinogen extravasation and the area of brain with vessels under axial stresses in excess of 200 kPa, equivalent to 0.14 axial strain considering the linear elastic model used for vessels. Shear stress and strain in the parenchyma have been suggested as predictors of BBB damage in previous studies^24,35–37^. For instance, it has been shown that the BBB breakdown in a rat model can occur at maximum principal logarithmic strains in the parenchyma larger than 0.19. However, these previous works lacked a description of vasculature, which limited their study to the correlation between stress and strain in parenchyma and BBB disruption. Using the multiscale finite element model and empirical measures of BBB disruption, we, for the first time, have provided evidence for the link between tensile stress in vessels and BBB damage. We have also suggested a value for axial stress and strain in vessels above which BBB breakdown may occur. Future work should extend this analysis to determine a threshold value for axial stress or strain in vessels for compromising BBB integrity.

We showed that the deviatoric stress in the parenchyma has a strong correlation with the tensile stress in vessels. In contrast, the total stress, which is the sum of the deviatoric stress and volumetric stress (= − hydrostatic pressure), has a weak correlation with stress in vessels. Brain tissue is nearly incompressible due to its high-water content but it is very compliant under shear loading^38,39^. These key biomechanical properties, which are incorporated in our multiscale models, manifest themselves in large shear deformations under small deviatoric stresses and very small volumetric deformations under large volumetric stresses^40,41^. These fundamental properties of the brain tissue can explain why deviatoric stress produces large deformation and stress in vessels. However, deviatoric stress cannot determine whether a vessel is under tension or compression, a state that depends on the angle between the stress direction and the axis of the vessel as shown by our microscale models.

This study is a step towards better understanding the interaction between the brain tissue and vasculature in traumatic brain injury. Here we assumed that vessels are tied to the surrounding brain tissue, thus undergoing the same deformation, in keeping with previous computational work^20,21,33^. This is a conservative assumption in the absence of experimental data that can provide better description for the interaction between vessels and parenchyma. We also used a linear elastic model for vessels based on previous work^20,27,42,43^. There is currently lack of experimental data on the mechanical behaviour of vessels, particularly for smaller vessels. Material characterisation of vessels at different length scales may improve the results of simulations and should be addressed in future works.

In this work we used the embedded element method to explicitly model the vasculature. This method has been used for explicit inclusion of both axonal fibres and vasculature in finite element models of brain. It has been shown that using this method for modelling axons results in underestimation of strain^44^, but this effect is likely to be small when incorporating vasculature due to their small volume fraction (5.4% in our model). Previous work has shown that using this method for modelling brain vasculature in human did not have a significant influence on the predicted strain in brain tissue^33^ but it reduced brain strain in some regions of interest by 36%^21^. In order to estimate the effects of incorporating vasculature in our model, we carried out CCI simulations with and without vasculature and compared the 95th percentile strains in the regions of interest shown in Fig. 4. We found negligible reduction in strain in the majority of regions (less than 1% in ROIs 2, 3 and 5). This reduction was larger in ROI 1 (18%) and ROI 4 (13%). This indicates that the explicit inclusion of vasculature may lead to a slightly stiffer response but considering the range of values of material properties of brain tissue and vessels often found in literature, this difference is negligible.

We used a CCI model in rats, which has limitations due to the differences between rat and human brains and loading conditions. Animal studies however provide novel information on the predictive capability of computational models of TBI and allow us to determine injury thresholds for biomechanical measures of tissue injury, such as stress and strain, which can be predicted in computational models of TBI in human regardless of the type of loading^17,22,45^. The validation of computational models is key to their reliable application and in order to do this, we need to eliminate as many confounding factors as possible. Hence the CCI model, which gives a better control over the biomechanics than closed head models, was chosen. Future studies on human, which include precise description of pathology and detailed reconstruction of the biomechanics of TBI would help in testing the determined injury thresholds and refining them for the use in predicting injury risk in human.

Our multiscale model is a unique tool that allows us to incorporate vascular anatomy and key biomechanical properties of the brain tissue in order to predict distribution of force and pathology in vasculature. This allows us to study the relationship between initial forces in small vessels, produced by head loading, and the progressive pathology in order to provide mechanistic links between head impacts and their acute and long-term effects post-trauma. The translation of this novel approach to the human allows us to test protection systems, such as helmets, for their effects on the prevention of vascular injuries and to guide their design improvements for more effective prevention of key TBI pathologies.

## Methods

**Figure 6:**
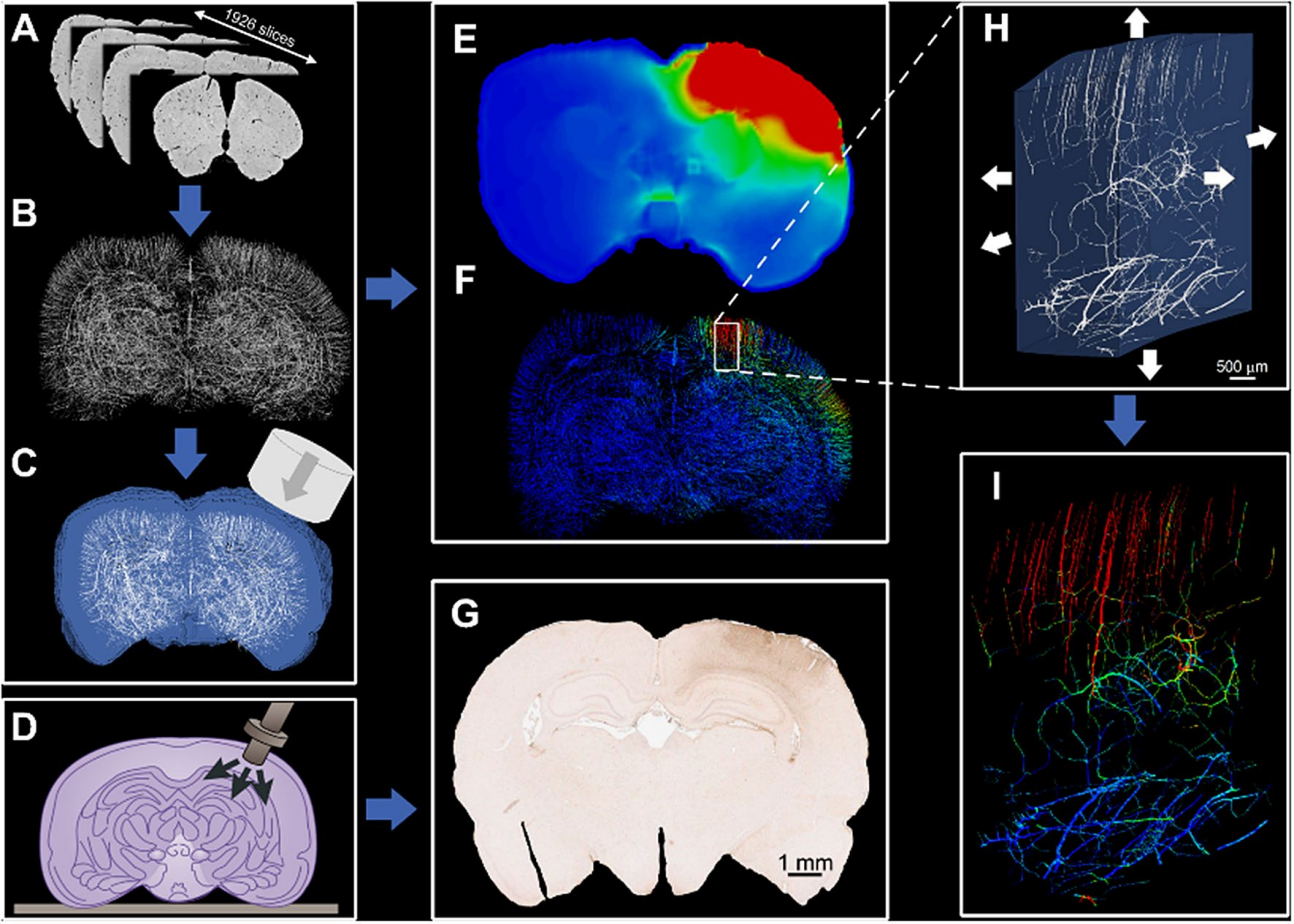
General workflow of this paper; (**A**) brain images acquired using SR-PCI technique, (**B**) FE model of the brain angioarchitecture, (**C**) FE model of CCI illustrating the impactor, brain tissue (blue) and vasculature (white), (**D**) schematic illustration of controlled cortical impact, (**E**) maximum value of the first principal strain of the brain tissue (red and blue colours show 50% and 0, respectively), (**F**) maximum axial stress within the vasculature (red and blue colours show 500 and 0 kPa respectively), (**G**) fibrinogen extravasation measured by immunohistochemistry (IHC) staining, (**H**) mapping the displacement at the borders of a region of interest for the microscale modelling, (**I**) vessels axial stress distribution of the microscale model of a region of interest (red and blue colours show 500 and 0 kPa respectively).

Figure 6 illustrates an overview of the methods, which are explained here.

### High-resolution 3D images of the brain

This part of our work has been published separately and we reused the images to generate a high-fidelity FE model. We briefly explain the method here and refer the readers to the original study published in Ref.^29^ for detailed information. Animal care and use was performed at Central South University, Changsha, China in accordance with the guidelines of the Administration Committee of Affairs Concerning Experimental Animals in Hunan Province, China. All experimental protocols were approved by the Animal Ethics Committee of Central South University, Changsha, China. A synchrotron radiation phase contrast imaging (SR-PCI) technique was used to develop an ultra-high-resolution 3D map of the brain angioarchitecture of a Sprague–Dawley rat. A BL13W1 beamline set-up (Shanghai Synchrotron Radiation Facility) featuring an electron storage ring with an accelerated energy of 3.5 GeV and an average beam current of 200 mA was used to extract the images. 1926 slices were obtained to extract 2D images for five samples, then 3D image of the brain was developed by registering 2D stacks of the images with the final resolution of 5.92 × 5.92 × 5.92 µm^3^. A commercially available software (Image Pro Analyser, Version 7.0, Media Cybernetics, Inc., USA, www.mediacy.com/imagepro) was used to reconstruct the 3D map of the angioarchitecture resulting in the reconstruction of vessels with a minimum diameter of 10 µm. The vessels’ diameters are between 10 to 100 μm (mean diameter is 17.85 μm). The mean vascular volume is 6118 μm^3^ and the vessels volume fraction (volume of vessels/volume of the brain) is 5.4%. After image registration, a unique map of the angioarchitecture was developed for all 5 animals. More details on the imaging technique and 3D reconstruction of the angioarchitecture is presented in^29^.

### Controlled cortical impact (CCI)

#### Animals and surgery

Animal care and use was performed at Imperial College London, UK in accordance with the ARRIVE guidelines under a personal licence (PIL), in compliance with a Home Office Project licence (PPL, granted to M. Sastre license number PFC130AFE granted on 05 November 2018) and the Animal (Scientific Procedures) Act 1986 and EU legislation. All experimental protocols were approved by the Animal Ethics Committee of Imperial College London, UK. Briefly, male Sprague Dawley rats were used, (9 weeks, Charles River, UK), housed in pairs on a 12 h light/dark cycle with ad-libitum access to standard rodent chow and water. The animals used for CCI are different from those used for SR-PCI imaging. General surgery and CCI procedure were carried out as previously described^46^ along with common data elements for CCI^47^. More details about surgical procedure is provided in Supplementary Material. 7 animals underwent surgery and CCI, and 3 animals underwent surgery only (naïve/sham). At 3 days post injury, all animals were subjected to terminal anaesthesia (Pentobarbital), followed by transcardial perfusion and tissue harvest. Brains were blocked, paraffin embedded, and serial coronal sections were cut from block 4 (− 3.12 mm).

#### Immunohistochemistry (IHC)

Paraffin sections were heated at 60 °C for 10 min, deparaffinised in xylene and rehydrated via decreasing ethanol concentrations (100%, 100% 90%, 70% and distilled H_2_O). Sections were permeabilised and endogenous peroxidase activity quenched in 1% H2O2 in PBS Triton X-100 (PBS-Tx) 0.3% for 30 min, followed by antigen retrieval with 0.01 M citrate buffer (pH 6) for 20 min in a steam bath. Sections were incubated overnight with primary antibody for anti-rabbit fibrinogen (Dako, Denmark, A0080, 1:2000) diluted in PBS-Tx 0.3% at 4 °C. The Super Sensitive Polymer-HRP IHC detection kit (BioGenex, USA) was used according to manufacturer’s protocol; Sections were counterstained with hematoxylin, then dehydrated with increasing concentrations of ethanol (70%, 90%, 100% and 100%) and xylene, and coverslipped with DPX.

#### Image acquisition and histology quantification

IHC sections were imaged at 20× resolution using the Aperio AT2 scanner and extracted as a TIF file with Aperio ImageScope software (Leica Biosystems, Germany). Fiji (ImageJ)^48^ was used to quantify fibrinogen extravasation. Regions of interest (ROIs) identified to examine for the ipsilateral and contralateral hemispheres included whole neocortex, neocortex underlying the CCI site (referred to as ‘dorsal’ neocortex), corpus callosum and hippocampus (Fig. 2C). The colour threshold value of positive fibrinogen staining was established at 155 and the percentage area coverage was measured. The colour threshold value was determined by measuring colour intensity which represented positive staining for fibrinogen extravasation and excluded background DAB (3,3′-diaminobenzidine) staining. Counterstaining was colour deconvoluted prior to quantitative analysis. Quantification of percentage area was conducted in three sections per animal and means were calculated.

### Finite element model

We segmented the stack of images described in 4.1 using thresholding technique in MATLAB to generate different parts for meshing. We segmented images based the intensity of voxels^49^. Then, we used an in-house MATLAB code to generate a voxel-based FE model of the brain tissue. The FE model was smoothed at the outer surface of the brain to avoid the effect of stress concentration at the impactor/brain interface. We did not observe any stress concentration at this surface or the interface between CSF/brain tissue, in agreement with our previous finite element study on rats^24^. In the macro scale model, a single solid element represents 4 voxels of the brain image to make the model less expensive, computationally. A separate in-house MATLAB code was developed to generate the FE model of the brain vasculature based on the 3D map of the angioarchitecture and assign the diameter of each point of the 3D map to the corresponding node of the FE model. Over 2,345,000 hexahedral solid elements with the size of 48 µm were used to discretise the brain tissue, and over 800,000 beam elements were used to discretise the angioarchitecture. We used beam elements with a circular cross section. Each beam was defined by two nodes at its ends. We used ELEMENT_BEAM_THICKNESS keyword in LS-Dyna^50^ to define the thickness of beams at each node, individually. This allowed us to generate beam elements with varying radii, matching the data presented in^29^.

The brain tissue was modelled as a hyper-viscoelastic material^24^. For the hyper-elastic part, the Ogden model with the following strain energy function was used:

1 $$\Psi ^{\infty } = \mathop \sum \limits_{{p = 1}}^{n} \frac{{\mu _{p} }}{{\alpha _{p} }}\left( {\lambda _{1}^{{\alpha _{p} }} + \lambda _{2}^{{\alpha _{p} }} + \lambda _{3}^{{\alpha _{p} }} - 3} \right),$$

where $\lambda _{{i~\left( {i = 1,~2~and~3} \right)}}$ are the principal stretches, and $\mu _{p}$ and $\alpha _{p}$ are material constants. The strain rate dependency of the tissue was incorporated into the model using the following equation:

2 $$S_{{\left( t \right)}} = S^{\infty } + \mathop \smallint \limits_{0}^{t} G\left( {t - T} \right)\frac{{\partial E\left( T \right)}}{{\partial T}}dT,$$

in which $S^{\infty }$ represents the long-term second Piola–Kirchhoff stress tensor, $E$ is the Green–Lagrange strain tensor and relaxation function $G\left( t \right)$ is represented by a Prony series:

3 $$G\left( t \right) = \mathop \sum \limits_{{i = 1}}^{n} G_{i} {\text{exp}}\left( {\frac{{ - t}}{{\tau _{i} }}} \right),$$

where $\tau _{i}$ and $G_{i}$ are material constants. The material constants for the brain tissue are given in the supplementary material (Table S1). The vasculature was modelled using a linear elastic constitutive model with an elastic modulus, Poisson’s ratio and density of 1.4 MPa, 0.38 and 104 kg/m^3^, respectively^20^.

We used “Automatic Surface to Surface” contact algorithm to define the contact at the contact interface between the impactor and the pia matter surface. The vasculature was tied to the surrounding tissue using “CONSTRAINED BEAM IN SOLID” keyword in LS-Dyna according to previous works^20,21,33^. This keyword provides constraint-based coupling between beams embedded in solid elements. In this approach, the degrees of freedom of the nodes of the beam elements are coupled to the nodes of the solid part in the neighbourhood of that node^50^. This approach allowed us to predict the axial stress in vessels.

To quantitatively compare the result of our simulations with histopathology, we mapped the axial stress of vessels to the brain tissue. An in-house code was developed to read the output file of LS-Dyna simulation to find the vessel elements in the neighbourhood of each tissue element. The code reads the centroid of each tissue element and each element of the vasculature. Then it finds the elements of the vasculature which are in the neighbourhood of each element of the brain tissue. We measured the distance between the centre of each vessel and tissue element, then the maximum axial stress of the vessel elements located in the neighbourhood of each element of the tissue was determined and assigned to that element (Fig. 2A,B). We calculated the area of the brain where the axial stress of the vessels exceeded 150, 200, 250 and 300 kPa across four anatomical regions of interests (i.e. corpus callosum, hippocampus, dorsal cortex and whole neocortex). We performed linear regression analysis between the area of fibrinogen extravasation and the brain area where vessels’ axial stress exceeded these values across mentioned anatomical ROIs.

After running the macro-scale simulation, we selected highly deformed regions of the brain as regions of interest for the micro-scale modelling. The selected regions where in the ipsilateral hemisphere and near the impactor, where we also observed maximal stress in the vessels. Mesh refinement was done for more accurate results at the micro scale and resulted in elements with minimum size of 20 µm. We used displacement-based boundary conditions^51^ at the border of regions of interests as shown in Fig. 6H. Then the model was set up to run individually and simulate the response of the tissue and the vessels within the regions of interest and produce results for further analysis.

A MATLAB code was developed to read the output of finite element analysis and quantify the interaction between the vessels’ direction and stress wave propagation within the tissue around the vessels. We calculated the direction of the angioarchitecture at each element of the angioarchitecture, the first principal stress, the first principal deviatoric stress of the tissue and its corresponding direction in the neighbourhood of each element of the vasculature. The vessels direction changes due to the deformation of the brain. We recorded the motion of the nodes of the beam elements representing the vessels at every 0.01 ms. This allowed us to determine the direction of the beam elements during brain deformation. We calculated the direction of each element of the angioarchitecture using the spatial coordinates of two nodes at two ends of each element at every 0.01 ms, as shown in Fig. 4C. For the elements in the neighbourhood of each element of the vasculature, we read the stress components of each element from the LS-Dyna output file and constructed the 3D stress matrix for each element. We calculated the eigen values and vectors of the stress matrix, which are principal stresses and their corresponding directions.

## Supplementary Information

Supplementary Information.
