# Supplementary material for: Multiscale modelling of cerebrovascular injury reveals the role of vascular anatomy and parenchymal shear stresses
Source: Sci Rep. 2021 Jun 21;11:12927. doi: 10.1038/s41598-021-92371-0 (PMC8217506; doi:10.1038/s41598-021-92371-0)
Supplement: Supplementary file 1 — Supplementary Information. [file 41598_2021_92371_MOESM1_ESM.docx]

**supplementary material for**

Multiscale modelling of cerebrovascular injury reveals the role of vascular anatomy and parenchymal shear stresses

Siamak Farajzadeh Khosroshahi*^1^, Xianzhen Yin^2^, Cornelius K. Donat^3,5^, Aisling McGarry^3^, Maria Yanez Lopez^3^, Nicoleta Baxan^4^, David J. Sharp^3^, Magdalena Sastre^3^, Mazdak Ghajari^1^.

1 Dyson School of Design Engineering, Imperial College London, London, UK

2 Shanghai Institute of Materia Medica

3 Department of Brain Sciences, Imperial College London, London, UK

4 Biological Imaging Centre, Imperial College London, London UK

5 Centre for Blast Injury Studies, Imperial College London, London, UK

* Corresponding author: Siamak Farajzadeh Khosroshahi.

**Email:**  [s.f.khosroshahi@imperial.ac.uk](mailto:s.f.khosroshahi@imperial.ac.uk)

**S.1. Surgical Procedures**

The experiments were carried out by authors working under a personal licence (PIL), in compliance with a Home Office Project licence (PPL, granted to M. Sastre) and the Animal (Scientific Procedures) Act 1986 and EU legislation and ARRIVE guidelines (Appendix A1) were adapted for all of the animal experiments.

Prior to surgery, 3dpi animals were randomized into two groups: sham-operation (n=4) and CCI (n=8). Anaesthesia was induced with 5% isoflurane (in oxygen) and maintained at 2-2.5%, with buprenorphine (0.05 mg/kg s.c.; Vetergesic, UK) administered as a perioperative analgesic at least 30 min before the first incision. The scalp was shaved, disinfected, and anaesthetized animals transferred to a stereotaxic frame. Body temperature was adjusted and maintained at 37°C using a rectal probe and a feedback-controlled heating pad.

All following surgical procedures were performed under aseptic conditions. Using additional local anaesthesia (Lidocaine, 1%; Mercury Pharma, Ireland), a midline incision was performed and the subcutaneous tissue and periosteum carefully retracted. A ~6 mm unilateral rectangular craniotomy, -0.5mm to -6.5 mm posterior and +3.5 mm lateral (centre) to Bregma was introduced. Any bleeding was controlled through topical application of adrenaline (0.075 mg/kg; Hameln, UK) soaked sterile gelatine sponges (Surgispon, Aegis Pharmaceuticals, India). Bone dust was removed with a surgical suction unit and the bone flap stored in sterile saline for later reimplantation. Injury was induced with a 5 mm flat electromagnetically driven steel impactor (23° angle), using the Leica Impact One (Leica Microsystems, UK). Impact on the dura was performed at 4 m/s for 100 milliseconds with an impact depth of 2 mm. Based on previous classification and our MRI and histology data, injury would be classified as moderate (1). Following impact, the dura was briefly covered with saline-soaked gelatine sponges to control potential epidural bleeding and inspected for signs of rupture. The bone flap was then re-implanted and the craniotomy closed with a nontoxic light-curing resin (Technovit 2200; Kulzer, Germany). Subcutaneous tissue and scalp were sutured with Vicryl Plus (5-0 and 4-0; Ethicon, UK). All rats received warmed saline (s.c.) and recovered under additional oxygen (10 min) and gentle warming. Following recovery, animals were single-housed, weighted and inspected twice daily for the first 72 hours and afterwards every morning. Buprenorphine was given every 12 hours (0.3 mg/kg, p.o.) for around 5 days, based on a scoring system (2). Following this period, rats were housed in the same pairs as before, with cage-mates subjected to the same type of procedure.

Three days post-impact, animals were subjected to a 9.4 T MRI scan. Aftwards, they were subjected to deep pentobarbital (Euthanal, Boehringer Ingelheim, UK) anaesthesia, followed by transcardial perfusion with ice-cold PBS with heparin (10 units/mL) and 4% PFA in PBS. Brains were post fixed for 24 hours in 4% PFA, stored in PBS (with 0.05% NaN3) at 4°C.

**S.2. Brain tissue material constants**

Table S.1 Constitutive material properties of the brain tissue (3).

| Tissue | Density [kg/m^3^] | $\mu_{1}$ [Pa] | $\alpha_{1}$ | $\mu_{2}$ [Pa] | $\alpha_{2}$ | Bulk modulus [MPa] |
| --- | --- | --- | --- | --- | --- | --- |
| Brain | 1040 | 29.5 | 10.1 | -66.0 | -12.9 | 50 |
| $\tau_{i}$ [ms] | $\tau_{1}$ = 0.001 | $\tau_{2}$ = 0.01 | $\tau_{3}$ = 0.1 | $\tau_{4}$ = 1 | $\tau_{5}$ = 10 | $\tau_{6}$ = 100 |
| $G_{i}$ [kPa] | $G_{1}$ = 175.5 | $G_{2}$= 42.8 | $G_{3}$ = 3.4 | $G_{4}$ = 4.4 | $G_{5}$= 0.05 | $G_{6}$ = 1.6 |

**References**

1. L. Siebold, A. Obenaus, R. Goyal, Criteria to define mild, moderate, and severe traumatic brain injury in the mouse controlled cortical impact model. *Exp. Neurol.* **310**, 48–57 (2018).

2. D. B. Morton, P. H. Griffiths, Guidelines on the recognition of pain, distress and discomfort in experimental animals and an hypothesis for assessment. *Vet. Rec.* **116**, 431–436 (1985).

3. C. K. Donat, *et al.*, From biomechanics to pathology: predicting axonal injury from patterns of strain after traumatic brain injury. *Brain* (2021) https:/doi.org/10.1093/brain/awaa336.

**Appendix A1**
